# Citric acid modified red mud for valorization as a sustainable catalyst in bisulfite-activated congo red degradation

**DOI:** 10.1038/s41598-025-20326-w

**Published:** 2025-10-21

**Authors:** Yonghua Huang, Cong Zhao, Shuai Liang, Zheng Wu, Daoping Peng, Yao Li, Yun Liu

**Affiliations:** 1 School of Environmental Science and Engineering, Southwest Jiaotong University, No. 999 Xi’an Road, Pidu District, Chengdu, 611756 Sichuan P. R. China; 2 School of Emergency Management, Xihua University, Chengdu, 610039 P.R. China; 3 Sichuan Jiucheng Testing Technology Co, Chengdu, 610000 P.R. China

**Keywords:** Red mud, Bisulfite, Advanced oxidation technology, Treating waste with waste, Co-pyrolysis, Pollution remediation, Pollution remediation, Environmental impact

## Abstract

Bisulfite (BS)-based advanced oxidation processes (AOPs) are attractive for pollutant degradation, but often depend on costly transition metals with leaching risks. Herein, we report a citric acid-modified red mud catalyst (RMAC) for efficient Congo Red (CR) removal. Citric acid acted Simultaneously as an acid activator and carbon template, enlarging the surface area from 31.10 to 116.40 m^2^ g^−1^ (3.74-fold increase). Under optimal conditions (5 mM BS, pH = 5, 80 mg L^−1^ CR), RMAC3-800 achieved 98.8% CR removal with a pseudo-first-order rate constant of 0.1399 min^−1^ and retained > 80% efficiency after three reuse cycles. Radical scavenging and EPR analyses confirmed SO_4_^•−^ (53.7%) and •OH (46.3%) as the dominant species, whereas XPS identified Fe^0^ as the principal active site. GC-MS detected six intermediates, supporting the proposed oxidative cleavage and mineralization pathways of the degradation process. A preliminary bench-scale cost analysis estimated an operating cost of ~ 13.94 RMB m^−3^ (≈ 1.95 USD m^−3^), underscoring its economic feasibility. This study demonstrates a cost-effective, recyclable, and sustainable catalytic system for wastewater treatment and red mud valorization.

**Supplementary Information:**

The online version contains supplementary material available at 10.1038/s41598-025-20326-w.

## Introduction

Azo dyes are extensively used in the textile, printing, paper, and leather industries because of their vivid colors, structural stability, and low production cost^1^. Among them, Congo Red (CR), a representative diazo dye, exhibits high water solubility and strong resistance to biodegradation, and its potential carcinogenicity and mutagenicity pose serious threats to ecosystems and human health^2^. Conventional treatment methods, including adsorption, membrane separation, biodegradation, and chemical oxidation, often suffer from limited efficiency, high operational costs, and the risk of secondary pollution^3^. Consequently, the development of efficient, economical, and environmentally sustainable strategies for CR degradation is urgently required.

Advanced oxidation processes (AOPs) have emerged as efficient strategies for degrading refractory pollutants through the in situ generation of reactive oxygen species (ROS), such as hydroxyl (•OH), sulfate (SO_4_^•−^), and superoxide (•O_2_^−^) radicals^4^. Compared with •OH, SO_4_^•−^ possesses a higher redox potential (2.5–3.1 V), longer lifetime, and greater stability across a wide pH range^5^. In practice, SO_4_^•−^ is commonly generated by activating peroxymonosulfate (PMS) or peroxydisulfate (PDS) using heat, ultraviolet irradiation, transition metals, heterogeneous catalysts, ultrasound, semiconductors, carbon-based materials, or electrochemical methods^6–8^. Despite their effectiveness, PMS/PDS-based systems often suffer from high reagent costs, limited catalyst stability, and potential secondary toxicity, which hinder their large-scale application^9,10^.

To overcome these limitations, sulfite (SO_3_^2−^) and bisulfite (BS, HSO_3_^−^) have attracted attention as abundant, inexpensive, and environmentally benign alternatives^11,12^. Under appropriate activation conditions, bisulfite can simultaneously produce •OH and SO_4_^•−^, thereby enabling the efficient degradation of organic pollutants^13,14^. Nevertheless, homogeneous activation with transition metal ions (e.g., Fe^2+^ and Co^2+^) suffers from narrow pH applicability, poor recyclability, and the risk of secondary contamination^15^. In contrast, heterogeneous catalysts offer more sustainable and reusable systems^16^. Thus, the development of efficient heterogeneous catalysts for bisulfite activation remains a critical challenge for practical applications.

Red mud (RM), a major solid byproduct of alumina production, is generated worldwide at 0.8–1.5 tons per ton of alumina^17^. Owing to its composition and abundance, RM has been explored for use in construction materials^18^, resource recycling^19^, and environmental remediation^20^. Its inherent porosity and surface area also make it a potential adsorbent for wastewater treatment^21^. Rich in metal oxides such as Fe_2_O_3_, TiO_2_, and Al_2_O_3_, RM is particularly attractive as a precursor for AOP catalysts, with iron species contributing to their catalytic activity^22,23^. However, unmodified RM suffers from low surface area, weak alkalinity, and poor catalytic performance, often leading to limited efficiency and secondary contamination^24^. Therefore, appropriate modification strategies are indispensable for enhancing their physicochemical and catalytic properties.

The co-pyrolysis of red mud with carbonaceous precursors has been widely explored to fabricate functional composites with enhanced surface area, porosity, and active site exposure, thereby extending their application in environmental remediation^25,26^. These composites generally exhibit improved catalytic activity and electron transfer capability, enabling the efficient degradation of dye pollutants in aqueous systems. For instance, the co-carbonization of red mud with waste sawdust increased the availability of Fe species and facilitated electron transfer, resulting in enhanced Fenton-like performance^27^. Similarly, Co_3_O_4_-decorated iron-containing biochar derived from the co-pyrolysis of red mud and spent coffee grounds showed the synergistic activation of peroxymonosulfate for effective dye degradation^28^. Collectively, these studies underscore co-pyrolysis as an efficient strategy for tailoring red-mud-based catalysts for advanced wastewater treatment.

Citric acid (CA), a biodegradable and non-toxic organic acid with multiple carboxyl groups and strong chelating ability, is an effective soft carbon source^29,30^. Acid treatment with CA can improve the surface properties and catalytic activity of red mud^31^. In addition, CA has been widely employed as a sacrificial template in fields such as concentrated solar power and lithium-ion battery production. Upon low-temperature decomposition, CA releases CO₂, promoting pore formation and thereby increasing the pore volume, pore size, and specific surface area^32,33^. These characteristics make CA a promising modifier for enhancing the physicochemical and catalytic performance of RM. However, the potential of CA-modified RM in bisulfite-based advanced oxidation processes remains largely uninvestigated.

This study presents a sustainable approach for converting industrial waste into functional catalysts for wastewater treatment. Citric acid-modified red mud catalysts (RMAC) were synthesized via an impregnation-co-pyrolysis strategy, and the effects of acid modification ratios and calcination temperatures on their physicochemical properties and catalytic performance were systematically investigated. The catalytic activity of RMAC in bisulfite-activated Congo Red degradation was evaluated, with an emphasis on key operating parameters and catalyst reusability. To elucidate the underlying mechanism, active sites and reactive species were probed through material characterization, radical quenching, and electron paramagnetic resonance (EPR) spectroscopy. Furthermore, GC-MS analysis identified degradation intermediates and enabled the proposal of plausible pathways, offering mechanistic insights and highlighting the practical potential of RMAC for wastewater remediation.

## Materials and methods

### Materials

Red Mud was obtained from a Bayer process residue dump in Zibo City, Shandong Province, China. The samples were ground through a 100-mesh Sieve, dried at 105 °C for 10 h, and stored in sealed polyethylene bags prior to use. Tert-butanol (TBA) and methanol (MeOH) were purchased from Aladdin Reagent Co., China. Anhydrous citric acid ((C_6_H_8_O_7_), sodium bisulfite (NaHSO_3_), sodium hydroxide (NaOH), hydrochloric acid (HCl), ethanol (EtOH), methylene blue, and Congo Red (CR) were obtained from Chengdu Kolon Chemical Reagent Co., China. Deionized water (18.2 MΩ·cm) was used to prepare all solutions.

### Preparation and characterization of citric acid-modified red mud-based catalyst

RMAC catalysts were synthesized via an impregnation-co-pyrolysis method. Red Mud and citric acid were mixed at mass ratios of 1:1, 3:1, and 5:1 in 40 mL of deionized water and stirred at room temperature for 4 h. The mixtures were dried at 65 °C for 72 h to obtain solid precursors, which were subsequently calcined in a tube furnace (BTF-1200 C-S) under N₂ at a Heating rate of 10 °C/min. The samples were maintained at the target temperatures (300, 500, or 800℃) for 2 h and then cooled to room temperature. After cooling, the products were washed with deionized water until a neutral pH was achieved and dried to yield the final catalysts. The samples were designated as RMACx-y, where *x* represents the citric acid/red mud mass ratio and *y* represents the calcination temperature. The point of zero charge (pH_pzc_) of RMAC3-800 was determined using the pH-drift method and measured as 6.1. The detailed characterization methods are provided in Text S1.

### Experimental procedures and analytical methods

To avoid overestimating the catalytic performance, adsorption-desorption pre-equilibration was conducted prior to BS addition. In a typical test, 100 mL of CR solution (initial concentration as specified) was placed in a beaker on a constant-temperature shaker (25 ± 2 °C, 200 rpm). The solution pH (3.0, 5.0, 7.0, or 9.0) was adjusted using HCl or NaOH and maintained throughout the experiment. Subsequently, 0.05 g of RMAC was added, and the suspension was kept in the dark for 30 min to reach adsorption-desorption equilibrium. Aliquots were withdrawn at designated intervals, filtered through 0.45 μm membranes, and analyzed at 497 nm (UV-4802 H spectrophotometer). The adsorption capacity was calculated using the following equation: (1) and (2)

1 $${{\text{q}}_{\text{t}}}= ({{\text{C}}_{{\text{0}}}}{\text{ - C)V/m}}$$

2 $$\:{\text{q}}_{\text{e}}\text=({\text{C}}_{\text{0}}\text{-}{\text{C}}_{\text{e}}\text{)V/m}\text{}$$

where C_0_, C, and C_e_ (mg L^−1^) are the CR concentrations at the initial time, time t, and equilibrium, respectively; V (L) is the solution volume; and m (g) is the mass of the RMAC.

After adsorption equilibrium (t = 0 for kinetics), BS was introduced at 5 mmol L^−1^ to initiate the reaction. The process was quenched with 0.1 mol L^−1^ Na_2_S_2_O_3_ solution. Pseudo-first-order kinetics were determined by fitting Eq. (3) to the data to obtain the apparent rate constant (*k*_*app*_, *min*^*−1*^) and the correlation coefficient (R^2^):

3 $$\:\text{Ln(C/}{\text{C}}_{\text{0}}\text{)=}{\text{}\text{-}\text{k}}_{\text{app}}\text{t}\text{}$$

The effects of key parameters, including BS dosage, pH, and initial CR concentration, on CR degradation by BS-activated RMAC3-800 were evaluated systematically. The catalyst reusability was assessed over three consecutive cycles. After each cycle, the reaction solution was collected and filtered, and the recovered RMAC3-800 was thoroughly washed with ethanol and deionized water, dried at 65 °C, and reused. The iron leaching in the solution was also analyzed after each cycle. To identify the dominant reactive oxygen species (ROS), tert-butanol (TBA) and methanol (MeOH) were employed as radical scavengers under identical conditions, and the corresponding CR removal efficiencies were determined. In addition, electron paramagnetic resonance (EPR) spectroscopy using DMPO (5,5-dimethyl-1-pyrroline-N-oxide) as a spin-trapping agent was used to detect ROS. The mineralization of CR was evaluated using total organic carbon (TOC) analysis with a TOC analyzer (TOC-L, Shimadzu, Japan). The detailed analytical procedures are provided in Text S2.

## Results and discussion

### Optimal Preparation conditions for RMAC catalyst

**Fig. 1:**
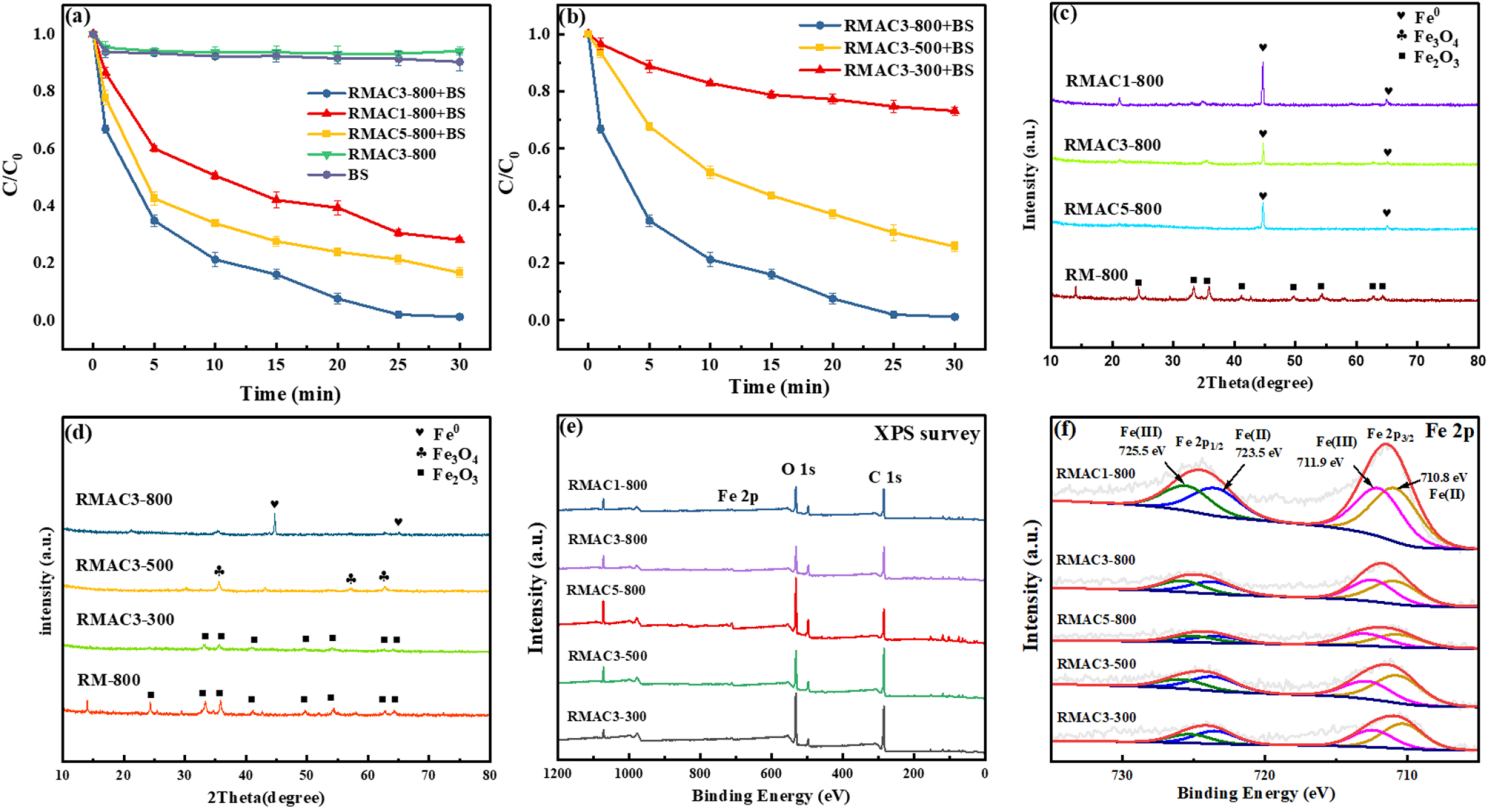
Removal of CR by RMACx-y prepared at different mass ratios (**a**) and pyrolysis temperatures (**b**); XRD patterns of RMACx-y prepared at different mass ratios (**c**) and pyrolysis temperatures (**d**); XPS spectra of RMACx-y at different mass ratios and pyrolysis temperatures: full spectrum (**e**), Fe 2p (f); Fig. 1(a) and (c): pyrolysis temperature = 800℃; Fig. 1(b) and (d): mass ratio of CA to RM = 3:1; Experimental conditions: pH = 5.0, [CR]_0_ = 80 mg L^−1^, RMAC catalyst dosage = 0.5 g L^−1^, [BS] = 5 mM.

To identify the optimal preparation conditions for red mud-based catalysts, the effects of the calcination temperature and CA loading on CR removal were systematically investigated. As shown in Figs. 1(a) and 1(b), increasing the CA-to-RM mass ratio from 1:1 to 3:1 markedly improved the removal efficiency from 71.9% to 98.8%, respectively. However, a further increase to 5:1 reduced the efficiency to 83.4%, likely because of the excessive organic content disrupting the catalyst structure. Similarly, elevating the calcination temperature from 300 to 800℃ progressively enhanced the catalytic activity, with the CR degradation rate increasing from 26.9% to 98.8%, suggesting that higher temperatures favor the formation of active sites. The catalyst prepared at a CA/RM ratio of 3:1 and calcined at 800℃—denoted as RMAC3-800—exhibited the best performance for BS-mediated CR degradation. To further elucidate their physicochemical properties, RMAC3-800 and related RMACx-y samples were characterized using X-ray diffraction (XRD), X-ray photoelectron spectroscopy (XPS), and Raman spectroscopy.

As shown in Figs. 1(c) and 1(d), the XRD patterns of RMACx-y indicate that the iron species in RM-800 primarily exist as Fe_2_O_3_ (JCPDS#33–0664) at 800℃. Upon citric acid modification, the characteristic diffraction peaks of Fe_2_O_3_ disappeared, while new peaks at 2θ = 44.6° and 65.0°, corresponding to metallic Fe^0^ (JCPDS#06-0696), emerged. This phase transformation is attributed to the thermal decomposition of citric acid, which generates reducing gases such as carbon monoxide (CO), thereby promoting the in situ reduction of Fe_2_O_3_. The stepwise reduction pathway can be expressed by Eqs. (4)-(6)^34^.

4 $$\:{\text{3F}}{{\text{e}}_{\text{2}}}{{\text{O}}_{\text{3}}}{\text{ + CO}} \to {\text{2F}}{{\text{e}}_{\text{3}}}{{\text{O}}_{\text{4}}}{\text{ + C}}{{\text{O}}_{\text{2}}}$$

5 $$\:{\text{Fe}}_{\text{3}}{\text{O}}_{\text{4}}\text{+CO}\rightarrow\text{3}\text{FeO}\text{+}{\text{CO}}_{\text{2}}$$

6 $$\:\text{FeO+CO}\rightarrow\text{}\text{Fe+}{\text{CO}}_{\text{2}}$$

As the calcination temperature increased from 300 to 500℃, the Fe_2_O_3_ content in RMACx-y decreased, accompanied by the formation of Fe_3_O_4_ (JCPDS#19–0629). At 800℃, Fe_3_O_4_ was completely reduced to Fe^0^ in RMAC3-500, and the intensity of the Fe^0^ peaks gradually declined with increasing citric acid loading, likely due to the enhanced reduction effect of CO released during pyrolysis^35^. These findings confirm a temperature-dependent sequential reduction of iron oxides, proceeding from Fe_2_O_3_ to Fe_3_O_4_ and ultimately to Fe^0^.

XPS analysis was performed to investigate the elemental compositions and chemical states of the RMACx-y samples. As shown in the survey spectrum (Fig. 1(e)), the RMAC was mainly composed of C, O, Fe, Al, Na, Si, Ti, and Ca. The binding energies at ~ 285.0, 530.0, and 711.0 eV correspond to the characteristic peaks of C 1 s, O 1 s, and Fe 2p, respectively.

The high-resolution Fe 2p spectrum (Fig. 1(f)) exhibits peaks at 710.8 and 723.5 eV, assigned to Fe^2+^ 2p3/2 and 2p1/2, and at 711.9 and 725.5 eV, attributed to Fe^3+^ 2p3/2 and 2p1/2, respectively^36^. With an increase in the CA/RM mass ratio from 1:1 to 5:1, the intensities of both Fe^2+^ and Fe^3+^ peaks decreased markedly, indicating that red mud acted as the primary iron source in the catalyst system^37^.

As shown in Fig. S1(a), three distinct peaks at 284.8, 286.6, and 289.3 eV correspond to the C-C/C = C, C-O, and C = O functional groups, respectively^38^. Among them, C-C and C = C bonds dominated, accounting for approximately 75%, whereas C = O contributed a smaller fraction. The peak intensities of these groups showed Little change with increasing citric acid dosage. In contrast, as the calcination temperature increased from 300 to 800℃, the intensities of all functional groups (C-C, C = C, C-O, and C = O) decreased markedly. This reduction is mainly attributed to the thermal decomposition of citric acid and other organic species, leading to the loss of surface functional groups at high temperatures^39^.

As shown in Fig. S1(b), the O 1 s spectrum exhibits peaks at 530.4, 531.9, and 533.7 eV, corresponding to the Fe-O, C-OH, and C = O species, respectively^40^. With increasing CA/RM mass ratio, the relative proportion of C = O increased from 5.66% to 13.61%, whereas that of Fe-O decreased from 14.43% to 7.81%. This shift is attributed to the incorporation of citric acid, which introduces additional oxygen-containing acidic groups^41^. The decrease in the Fe-O content reflected a reduced contribution from red Mud, further confirming its role as the primary iron source. Moreover, as the calcination temperature increased from 300 to 800℃, both the intensity and proportion of Fe-O decreased Significantly. Together with the Fe 2p XPS results, these observations indicate a stepwise reduction of iron oxides to Fe^0^ during pyrolysis, which is consistent with the XRD findings.

Raman spectroscopy provides valuable insight into the carbon structure of catalysts. As shown in Fig. S1(c), RMACx-y samples exhibited two characteristic peaks: the D band (~ 1330 cm^−1^), associated with structural defects and sp^3^-hybridized (amorphous) carbon, and the G band (~ 1590 cm^−1^), corresponding to sp^2^-hybridized graphitic carbon^42–44^. The intensity ratio I_D_/I_G_ is commonly used to assess the defect density, with higher values indicating greater disorder^45^. With increasing pyrolysis temperature, the I_D_/I_G_ ratio increased, suggesting that higher temperatures promote carbon disorder and the formation of defects. Among all the samples, RMAC3-800 displayed the highest I_D_/I_G_ value, indicating the most disordered carbon structure at a CA/RM ratio of 3:1 and 800℃. This enhanced defect density is expected to provide additional active sites for reactive oxygen species, thereby facilitating BS activation and improving CR degradation efficiency^46^.

### Characterization of RMAC3-800 under optimal Preparation conditions

**Fig. 2:**
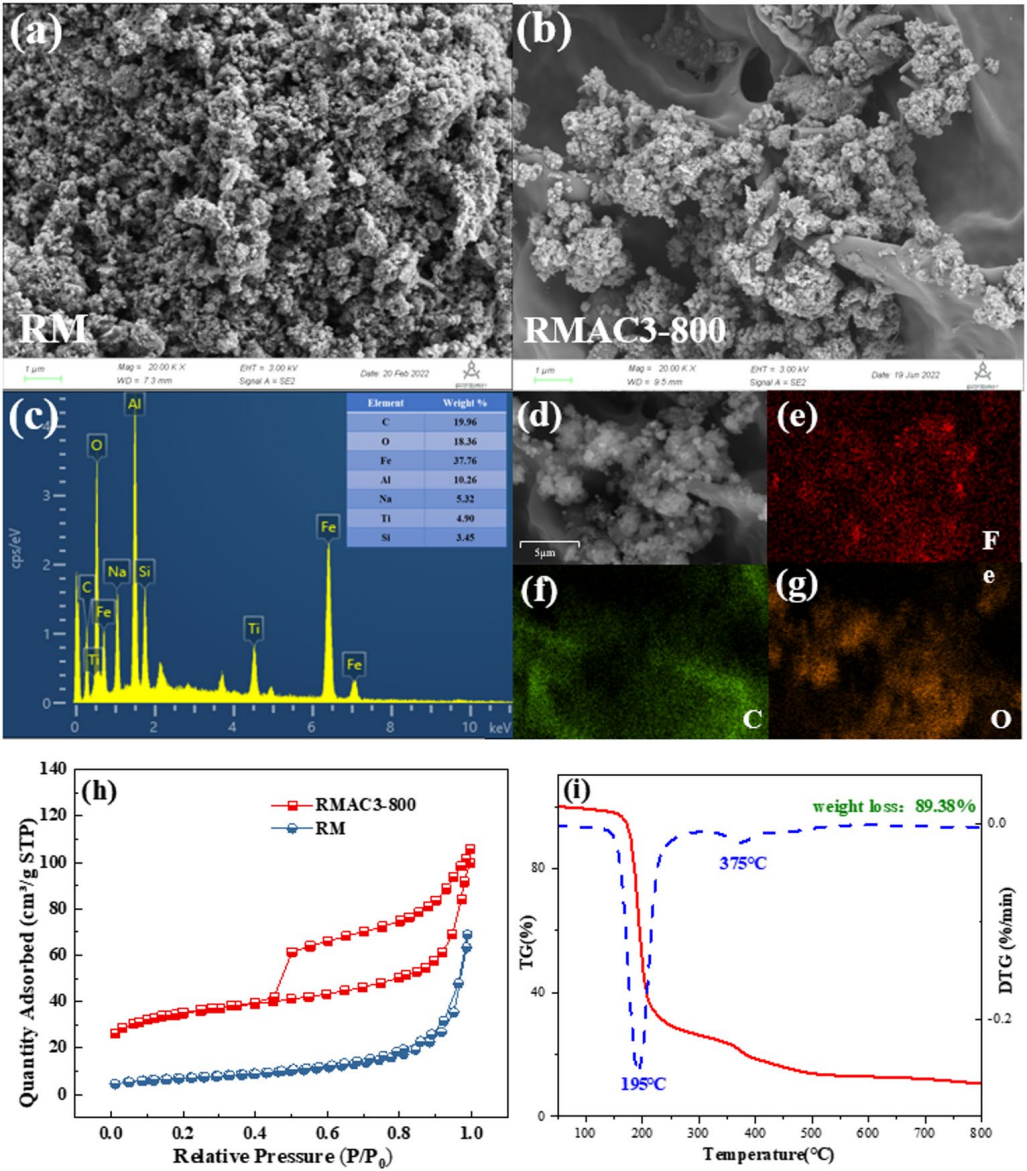
SEM images of RM (**a**) and RMAC3-800 (**b**); EDS energy spectra (**c**) and elemental distributions (**d**-**g**) of RMAC3-800; N_2_ adsorption-desorption isotherms of RM and RMAC3-800 (**h**); TG and DTG curves of RMAC3-800 preparation (**i**).

Scanning electron microscopy (SEM) and energy-dispersive spectroscopy (EDS) were used to examine the morphology and elemental distribution of the samples (Fig. 2(a-g)). As shown in Fig. 2(a), raw red mud consists of irregularly sized agglomerated particles with a loosely porous structure^47^. In contrast, RMAC3-800 exhibited a more defined and interconnected porous network. EDS mapping (Fig. 2(e)) further revealed a more uniform distribution of Fe across the RMAC3-800 surface. This improvement is attributed to citric acid pyrolysis, which generates gases that act as pore-forming agents and carbon templates, thereby promoting uniform particle dispersion and increasing the surface area. The resulting porous structure provides a greater number of accessible catalytic sites^47^. EDS analysis also confirmed that RMAC3-800 was mainly composed of Fe, C, O, Al, Na, Ti and Si.

As shown in Fig. 2(h), the N₂ adsorption-desorption isotherms of RM-800 and RMAC3-800 reveal distinct textural differences. RM-800 exhibited a Type III isotherm, indicative of a non-porous or macroporous structure, whereas RMAC3-800 displayed a Type IV isotherm with an H3-type hysteresis loop, characteristic of slit-like mesopores^48^. BET analysis (Table S1) showed that the specific surface area of RMAC3-800 increased 3.74-fold to 116.40 m^2^ g^−1^ after citric acid modification, confirming the activation effect of citric acid^49^. The pore volume and average pore diameter also increased Significantly, reaching 0.151 cm^3^ g^−1^ and 51.91 nm, respectively. These enhancements suggest that the carbon template generated during citric acid pyrolysis markedly improved the porous structure and surface area, thereby providing additional active sites for CR adsorption and degradation, consistent with the SEM observations.

To further investigate the physicochemical changes during pyrolysis, thermogravimetric (TG) analysis of RMAC3-800 was performed (Fig. 2(i)). The total weight loss reached 89.38%, with two distinct stages observed at ~ 195 and 375℃. The major weight loss at 195℃ is attributed to citric acid decomposition, accompanied by the evaporation of crystallization water and the release of gases such as CO, CO_2_, and H_2_O, which is consistent with the C 1 s XPS results^35^. The smaller loss at 375℃ likely corresponds to the removal of chemisorbed water from the red mud surface^50,51^.

The magnetic properties of RMAC3-800 are presented in Fig. S3. The magnetization curve exhibited a nonlinear response with negligible residual magnetization and coercivity, indicating superparamagnetic behavior of the nanocomposites. With increasing magnetic field strength, the magnetization gradually saturated at 19.30 emu g^−1^. This strong magnetism facilitates the easy recovery and reuse of RMAC3-800, underscoring its potential for practical applications^52^.

### Study on the effect of RMAC3-800-activated BS in degrading CR

The overall effectiveness of the RMAC3-800/BS system in degrading CR is illustrated in Fig. S2, showing a remarkable decolorization of the dye solution under the optimized conditions. To quantitatively assess the cooperative effect between RMAC3-800 and BS, pseudo-first-order kinetic analyses were performed for RMAC3-800 alone, BS alone, and their combination (Fig. S4). The synergistic effect (SE) was calculated using Eq. (7)^53^:

7 $$\:\text{SE=}\frac{{\text{k}}_{\text{app,RMAC3-800+BS}}}{{\text{k}}_{\text{app,RMAC3-800}}\text{+}{\text{k}}_{\text{app,BS}\text{}}}\text{}\text{}\text{}$$

where *k*_*app, RMAC3−800+BS*_, *k*_*app, RMAC3−800*_, *and k*_*app, BS*_ represent the apparent rate constants for the combined, catalyst-only, and BS-only systems, respectively. The calculated values of 0.1399, 0.0013, and 0.0022 min^−1^ yielded an SE of 39.97. An SE value Significantly greater than 1 confirmed a strong synergistic interaction between RMAC3-800 and BS. This enhanced activity can be attributed to the concurrent presence of Fe species in RMAC3-800, which efficiently promoted BS activation^54^.

To distinguish between adsorption and catalytic oxidation, adsorption-desorption experiments were performed prior to BS addition. As shown in Fig. 1(a), RMAC3-800 exhibited an adsorption capacity of 1.09 mg g^−1^, corresponding to an equilibrium removal of 6.84% after 30 min of contact time. In contrast, the RMAC3-800/BS system removed nearly all CR within the same period, demonstrating that adsorption accounted for only a minor fraction of the total removal and that oxidative degradation was the dominant pathway.

As shown in Fig. 4(a), the CR removal efficiency of the RMAC3-800/BS system was strongly pH-dependent. The efficiency increased from pH 3.0 to 5.0, reaching 98.8%, but declined sharply under neutral and alkaline conditions. This behavior can be explained by the combined effects of the surface charge and solution chemistry. The pH_pzc_ of RMAC3-800 was determined to be 6.1; thus, at pH < pH_pzc_, the positively charged catalyst surface promotes the electrostatic attraction of bisulfite anions and facilitates their activation. In contrast, at pH_pzc_, electrostatic repulsion dominates and suppresses reactivity^55^. Acidic conditions also accelerate Fe^0^ dissolution and Fe^2+^ generation, whereas excessively low pH favors SO_2_ formation, thereby reducing the radical yield^56,57^ (Eqs. (8)-(13)). Under alkaline conditions, OH^−^ can react with SO_4_^2−^ to produce •OH (Eq. (14)). However, the lower redox potential of •OH compared to SO_4_^•−^ and its self-quenching with OH^−^ (Eqs. (14)-(15)) lead to diminished degradation efficiency^58,59^.

**Fig. 3:**
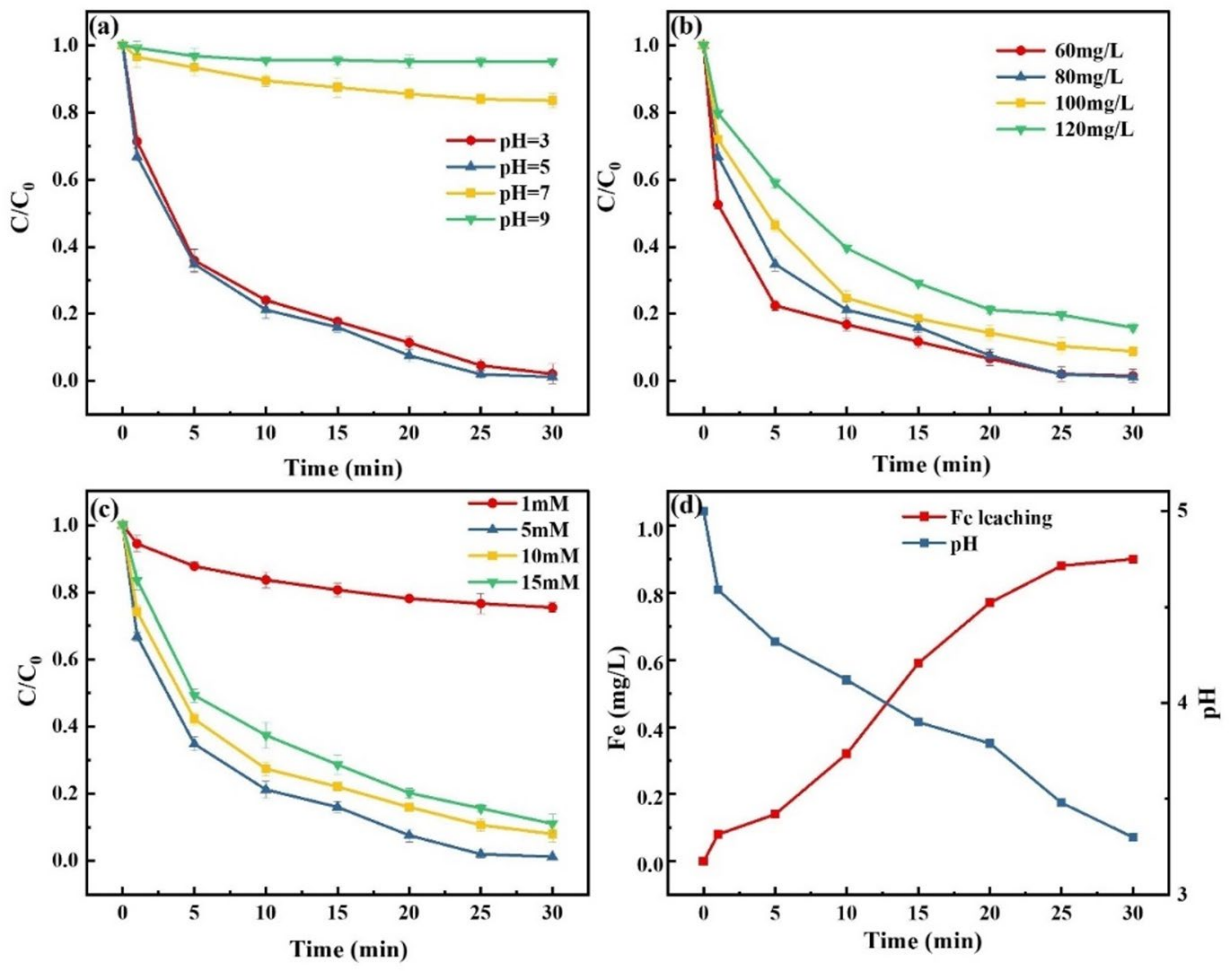
Effect of different pH (**a**), initial CR concentration (**b**), and BS dosage (**c**) on the removal effect of CR; Change curves of pH and Fe leaching during the reaction (**d**); Fig. 3(a): [CR]_0_ = 80 mg L^−1^, [BS] = 5 mM; Fig. 3(b): pH = 5.0, [BS] = 5 mM; Fig. 3(c): pH = 5.0, [CR]_0_ = 80 mg L^−1^; Experimental conditions: RMAC3-800 catalyst dosage = 0.5 g L^−1^, pyrolysis temperature = 800℃, mass ratio of CA to RM = 3:1.

As shown in Fig. 3(d), the solution pH decreased sharply within the first 5 min and then declined more gradually, indicating progressive acidification of the reaction system. Concurrently, the Fe concentration in the solution increased, suggesting the continuous transformation of Fe^0^ to Fe^2+^. Together with the results shown in Fig. 3(a), these findings confirm that acidic conditions favor CR degradation. Despite Fe leaching, the maximum Fe concentration remained below 0.9 mg L^−1^, demonstrating that heterogeneous Fe species on the catalyst surface remained the dominant contributors to the reaction.

The effect of the initial CR concentration on the degradation performance was also examined. As shown in Fig. 3(b), increasing the CR concentration from 60 to 120 mg L^−1^ gradually reduced the removal efficiency, mainly because the fixed dosages of the catalyst and BS Limited the generation of reactive radicals. Nevertheless, a removal efficiency of 84.2% was achieved at 120 mg L^−1^, highlighting the strong degradation capacity of the RMAC/BS system and its adaptability to different pollutant loads.

The effect of BS dosage on CR removal is shown in Fig. 3(c). Increasing the BS concentration from 1 to 5 mM Significantly enhanced the removal efficiency from 24.5% to 98.8%, owing to the greater generation of reactive oxygen species with higher BS availability. However, further increases in BS dosage caused a gradual decline in the efficiency. This decrease can be attributed to the saturation of the active sites on the catalyst surface and radical quenching reactions at excessive BS levels (Eqs. (16)-(19)), where surplus radicals preferentially interact with each other rather than with CR, leading to BS overconsumption and a reduced degradation efficiency^60,61^.

8 $$\:{\text{Fe}}^{\text{0}}\rightarrow{\text{Fe}}^{\text{2+}}\text{+}{\text{2e}}^{\text{-}}$$

9 $$\:{\text{F}}{{\text{e}}^{{\text{2 + }}}}{\text{ + HS}}{{\text{O}}_{\text{3}}}^{\text{-}} \to {\text{FeHS}}{{\text{O}}_{\text{3}}}^{\text{ + }}$$

10 $$\:{\text{4FeHS}}{{\text{O}}_{\text{3}}}^{\text{ + }}{\text{ + }}{{\text{O}}_{\text{2}}} \to {\text{4FeS}}{{\text{O}}_{\text{3}}}^{\text{ + }}{\text{ + 2}}{{\text{H}}_{\text{2}}}{\text{O}}$$

11 $$\:{\text{FeS}}{{\text{O}}_{\text{3}}}^{\text{ + }}{\text{ + 2}}{{\text{H}}_{\text{2}}}{\text{O}} \to {\text{F}}{{\text{e}}^{{\text{2 + }}}}{\text{ + S}}{{\text{O}}_{\text{3}}}^{ \bullet {\text{ - }}}$$

12 $$\:{\text{S}}{{\text{O}}_{\text{3}}}^{ \bullet {\text{ - }}}{\text{ + }}{{\text{O}}_{\text{2}}} \to {\text{S}}{{\text{O}}_{\text{5}}}^{ \bullet {\text{ - }}}$$

13 $${\text{S}}{{\text{O}}_{\text{3}}}^{ \bullet {\text{ - }}}{\text{ + HS}}{{\text{O}}_{\text{3}}}^{\text{-}} \to {\text{S}}{{\text{O}}_{\text{4}}}^{ \bullet {\text{ - }}}{\text{ + S}}{{\text{O}}_{\text{4}}}^{{\text{2 - }}}{\text{ + }}{{\text{H}}^{\text{ + }}}$$

14 $$\:{\text{S}}{{\text{O}}_{\text{4}}}^{ \bullet {\text{ - }}}{\text{ + O}}{{\text{H}}^{\text{-}}} \to {\text{O}}{{\text{H}}^ \bullet }{\text{ + S}}{{\text{O}}_{\text{4}}}^{{\text{2 - }}}$$

15 $$\:{\text{O}}{{\text{H}}^ \bullet }{\text{ + O}}{{\text{H}}^{\text{-}}} \to {{\text{O}}^{ \bullet {\text{ - }}}}{\text{ + }}{{\text{H}}_{\text{2}}}{\text{O}}$$

16 $$\:{\text{S}}{{\text{O}}_{\text{4}}}^{ \bullet {\text{ - }}}{\text{ + HS}}{{\text{O}}_{\text{3}}}^{\text{-}} \to {\text{S}}{{\text{O}}_{\text{3}}}^{ \bullet {\text{ - }}}{\text{ + S}}{{\text{O}}_{\text{4}}}^{{\text{2 - }}}{\text{ + }}{{\text{H}}^{\text{ + }}}$$

17 $$\:{\text{O}}{{\text{H}}^ \bullet }{\text{ + HS}}{{\text{O}}_{\text{3}}}^{\text{-}} \to {{\text{H}}_{\text{2}}}{\text{O + S}}{{\text{O}}_{\text{3}}}^{ \bullet {\text{ - }}}$$

18 $$\:{\text{S}}{{\text{O}}_{\text{4}}}^{ \bullet {\text{ - }}}{\text{ + S}}{{\text{O}}_{\text{4}}}^{ \bullet {\text{ - }}} \to {{\text{S}}_{\text{2}}}{{\text{O}}_{\text{8}}}^{{\text{2 - }}}$$

19 $$\:{\text{S}}{{\text{O}}_{\text{4}}}^{ \bullet {\text{ - }}}{\text{ + }}{{\text{S}}_{\text{2}}}{{\text{O}}_{\text{8}}}^{{\text{2 - }}} \to {\text{S}}{{\text{O}}_{\text{4}}}^{{\text{2 - }}}{\text{ + }}{{\text{S}}_{\text{2}}}{{\text{O}}_{\text{8}}}^{ \bullet {\text{ - }}}$$

**Fig. 4:**
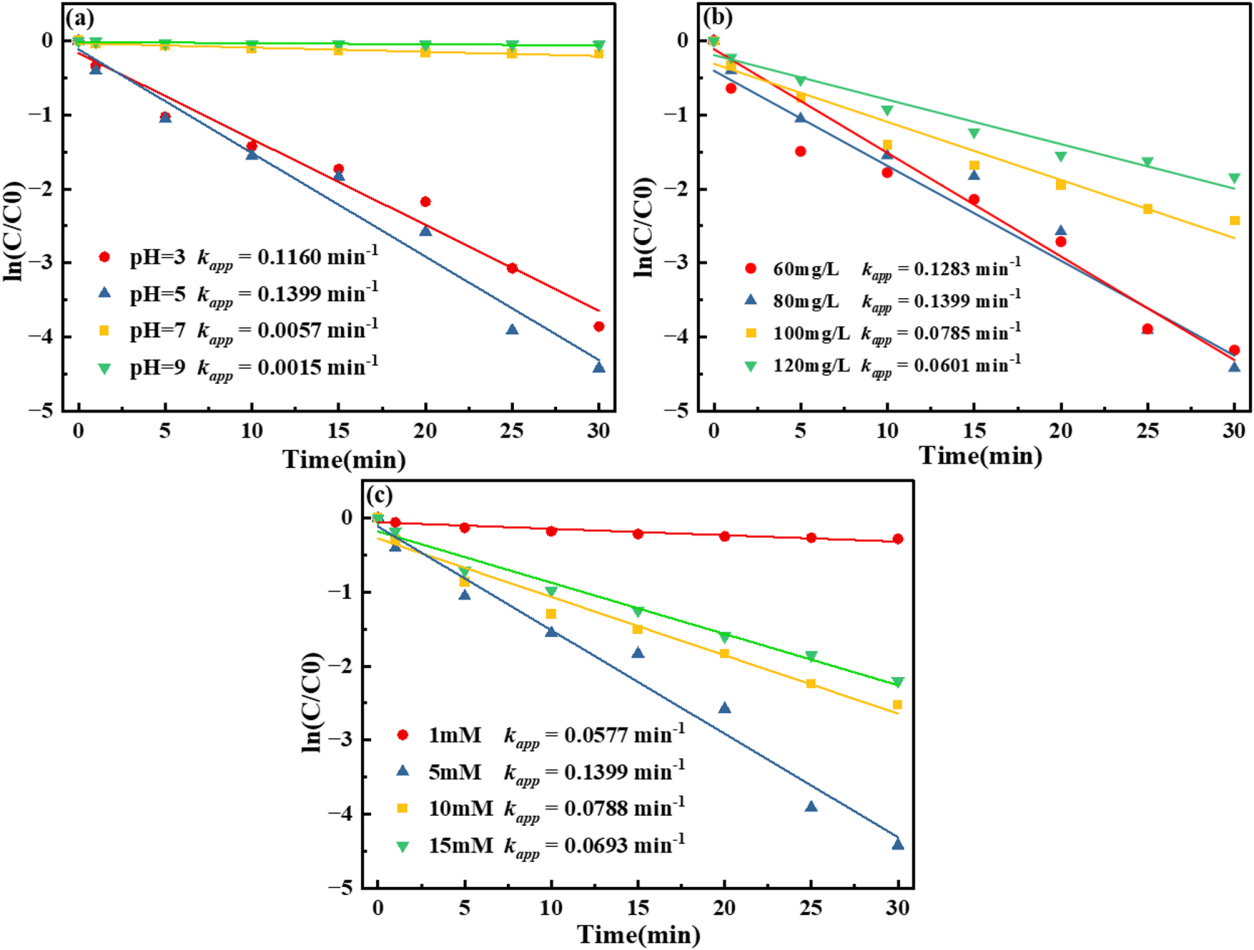
Pseudo-first-order kinetic fitting of CR degradation by the RMAC3-800/BS system under different pH values(**a**), initial CR concentrations (**b**), and BS dosages(**c**).

The degradation of CR by the RMAC3-800/BS system at varying pH, BS dosages, and initial CR concentrations followed a pseudo-first-order kinetic model (Fig. 4), with correlation coefficients (R^2^) generally above 0.90, indicating good linearity. The apparent rate constants (*k*_*app*_) varied considerably with the reaction conditions: acidic pH and moderate BS dosage accelerated degradation, whereas neutral or alkaline pH, excessive BS, and higher pollutant concentrations suppressed the rates. The maximum *k*_*app*_ of 0.1399 min^−1^ (R^2^ = 0.9741) was achieved at pH 5 with 5 mM BS and 60 mg L^−1^ CR, representing the optimal conditions for CR removal in the RMAC3-800/BS system.

### Reusability of RMAC3-800

**Fig. 5:**
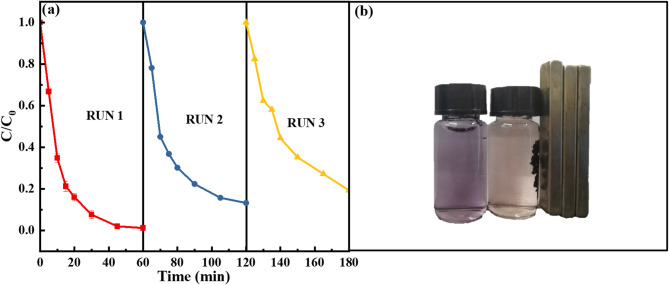
Reusability of RMAC3-800 (**a**), recycling effect (**b**); Experimental conditions: pH = 5.0, [CR]_0_ = 80 mg L^−1^, RMAC catalyst dosage = 0.5 g L^−1^, [BS] = 5 mM, pyrolysis temperature = 800℃, mass ratio of CA to RM = 3:1.

**Fig. 6:**
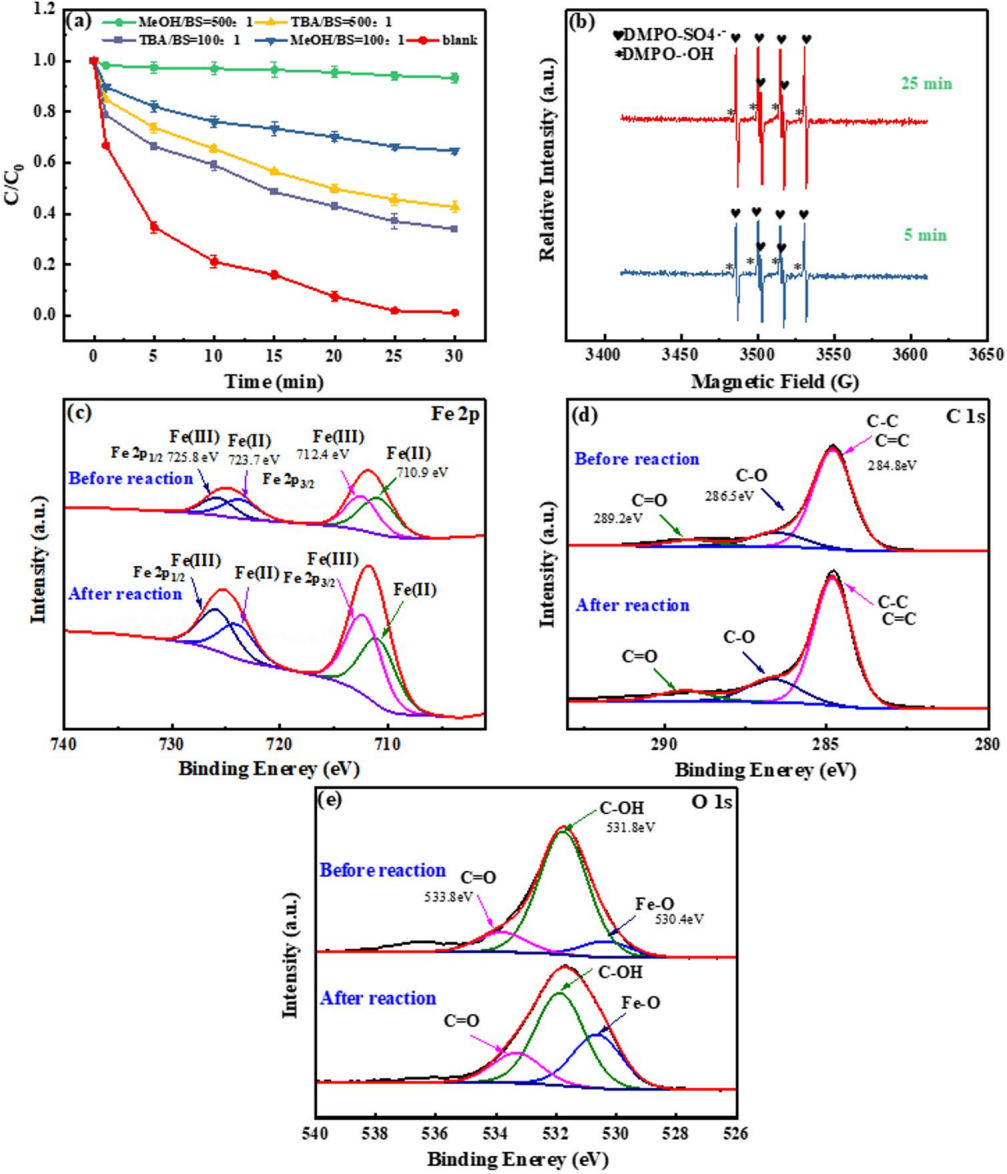
Effect of two quenchers (MeOH and TBA) on CR degradation (**a**); EPR spectra of free radicals in the DMPO capture system (**b**); XPS spectra before and after the reaction of RMAC3-800: Fe 2p (**c**), C 1 s (**d**), and O 1 s (**e**); Experimental conditions: pH = 5.0, Initial CR concentration = 80 mg L^−1^, RMAC catalyst dosage = 0.5 g L^−1^, BS concentration = 5 mM, pyrolysis temperature = 800℃, mass ratio of CA to RM = 3:1.

To evaluate the reusability of RMAC3-800 under practical conditions, three consecutive recycling experiments were conducted (Fig. 5(a)), and the recovery process is shown in Fig. 5(b). The CR removal efficiency declined slightly to 86.9% in the second cycle and further to 80.9% in the third cycle, indicating a modest loss of catalytic activity. This decrease is mainly attributed to Fe dissolution and the accumulation of degradation intermediates on the catalyst surface, which hinders the interaction between BS and the catalyst^62^. In the sulfite activation system, the leached species are predominantly Fe^2+^, which is partially oxidized to Fe^3+^ under acidic conditions, forming an Fe^2+^/Fe^3+^ redox cycle that sustains radical generation. However, the reduction of Fe^3+^ to Fe^2+^ is kinetically limited, restricting Fe^2+^ regeneration and preventing the cycle from completing spontaneously^63^. This interpretation is supported by the XPS results (Fig. 6), which show a decrease in Fe^2+^ and an increase in Fe^3^⁺ after reaction. In addition, the strong magnetic properties of RMAC3-800, as confirmed by the VSM (vibrating sample magnetometer) results in Sect. 3.2 (Fig. S3), enabling easy recovery, further demonstrating its good recyclability and potential for practical application.

### Mechanistic investigation of CR degradation by the RMAC3-800/BS system

#### Detection of reactive oxygen species produced by RMAC3-800 activated BS

To identify the reactive oxygen species (ROS) involved and clarify the degradation mechanism of the RMAC3-800/BS system, radical quenching experiments were performed using MeOH and TBA. MeOH quenches both SO_4_^•−^ and •OH, whereas TBA selectively quenches •OH^64^. As shown in Fig. 6(a), the addition of MeOH markedly suppressed CR removal, with the efficiency decreasing to 35.4% at a MeOH/BS ratio of 100:1 and further to 6.67% at 500:1, indicating that both SO_4_^•−^ and •OH are major contributors to the degradation process. In contrast, TBA caused partial inhibition, reducing CR removal to 64.8% and 56.2% at TBA/BS ratios of 100:1 and 500:1, respectively. Based on these results (Table S2), the relative contributions of SO_4_^•−^ and •OH were estimated to be 53.7% and 46.3%, respectively, suggesting that both ROS play comparable roles in CR degradation.

To further verify the generation of SO_4_^•−^ and •OH in the RMAC3-800/BS system, EPR spectroscopy was performed using DMPO as a spin-trapping agent. As shown in Fig. 6(b), characteristic signals of both DMPO-•OH and DMPO- SO_4_^•−^ were observed, confirming the formation of these species. In addition, the signal intensities increased with reaction time, indicating the continuous generation of SO_4_^•−^ and •OH during CR degradation by BS-activated RMAC3-800^65^.

The MeOH/TBA quenching and DMPO-EPR results confirmed that SO_4_^•−^ and •OH were the dominant reactive species in the RMAC3-800/BS system. Nevertheless, previous studies on red-mud-based catalysts have also reported the generation of O_2_^•−^/HO_2_• and ^1^O_2_ in BS/S(IV) activation^66–68^. Thus, the possible contributions of these species cannot be excluded and merit further investigations.

#### Probing the active site of RMAC3-800

To further elucidate the activation mechanism of BS by RMAC3-800, XPS analyses of Fe 2p, C 1 s, and O 1 s were conducted on the catalyst surface before and after the reaction. As shown in Fig. 6(c), the Fe 2p spectrum displayed peaks corresponding to Fe^2+^ (710.9 and 712.4 eV) and Fe^3+^ (723.7 and 725.8 eV)^69^, while no distinct Fe^0^ signal was detected on the surface. This indicates that Fe^0^ is primarily embedded within the internal pores rather than being exposed on the outer surface. During the reaction, Fe^0^ gradually releases Fe^2+^, which is subsequently oxidized to Fe^3+^, thereby sustaining the electron transfer and radical generation. This interpretation reconciles the bulk-sensitive XRD detection of Fe^0^ with the surface-sensitive XPS results, confirming that Fe^0^ is a crucial internal active site.

Comparison of the Fe 2p spectra before and after reaction revealed that the proportion of Fe^3+^ increased from 46.1% to 54.6%, while Fe^2+^ decreased from 53.9% to 45.4%, indicating the oxidation of Fe^2+^ to Fe^3+^ during the degradation process (Eq. (20))^70^. In addition, the overall intensity of the Fe 2p peaks after the reaction was ~ 2.1 times higher than that before, suggesting that electron transfer occurred and that Fe^0^ continuously released Fe^2+^ throughout the reaction.

As shown in Fig. 6(d), the C 1 s spectrum exhibited peaks at 284.8, 286.8, and 289.2 eV, corresponding to the C-C/C = C, C-O, and C = O groups, respectively^71^. After the reaction, the proportion of C-O species increased from 14.71% to 18.81%, whereas C-C/C = C decreased from 76.4% to 72.75%, indicating the partial oxidation of carbon species on the catalyst surface during BS activation.

Previous studies have shown that pollutant degradation by catalyst-activated PS often involves the oxidation of non-oxygenated functional groups to oxygenated ones^72^. Accordingly, during BS activation by RMAC3-800, non-oxygenated groups such as C-C and C = C are Likely oxidized to oxygenated groups such as C-O. As shown in the O 1s spectrum (Fig. 6(e)), the peaks at 530.4, 531.8, and 533.8 eV correspond to the Fe-O, C-OH, and C = O species, respectively^73^. After the reaction, the proportion of Fe-O increased markedly from 9.96% to 30.46%, consistent with the enhanced Fe 2p peak intensity, further confirming the electron-transfer role of Fe^0^. In addition, the proportion of C = O increased by 36.4% (from 12.51% to 17.06%), indicating that the degradation process involved the transformation of non-oxygenated groups into oxygenated groups.

20 $$\:{\text{S}}{{\text{O}}_{\text{5}}}^{ \bullet {\text{ - }}}{\text{ + 2F}}{{\text{e}}^{{\text{2 + }}}}{\text{ + }}{{\text{H}}_{\text{2}}}{\text{O}} \to {\text{2F}}{{\text{e}}^{{\text{3 + }}}}{\text{ + S}}{{\text{O}}_{\text{4}}}^{ \bullet {\text{ - }}}{\text{ + O}}{{\text{H}}^{\text{-}}}$$

#### Product and pathway analysis of CR degradation by the RMAC3-800/BS system

**Fig. 7:**
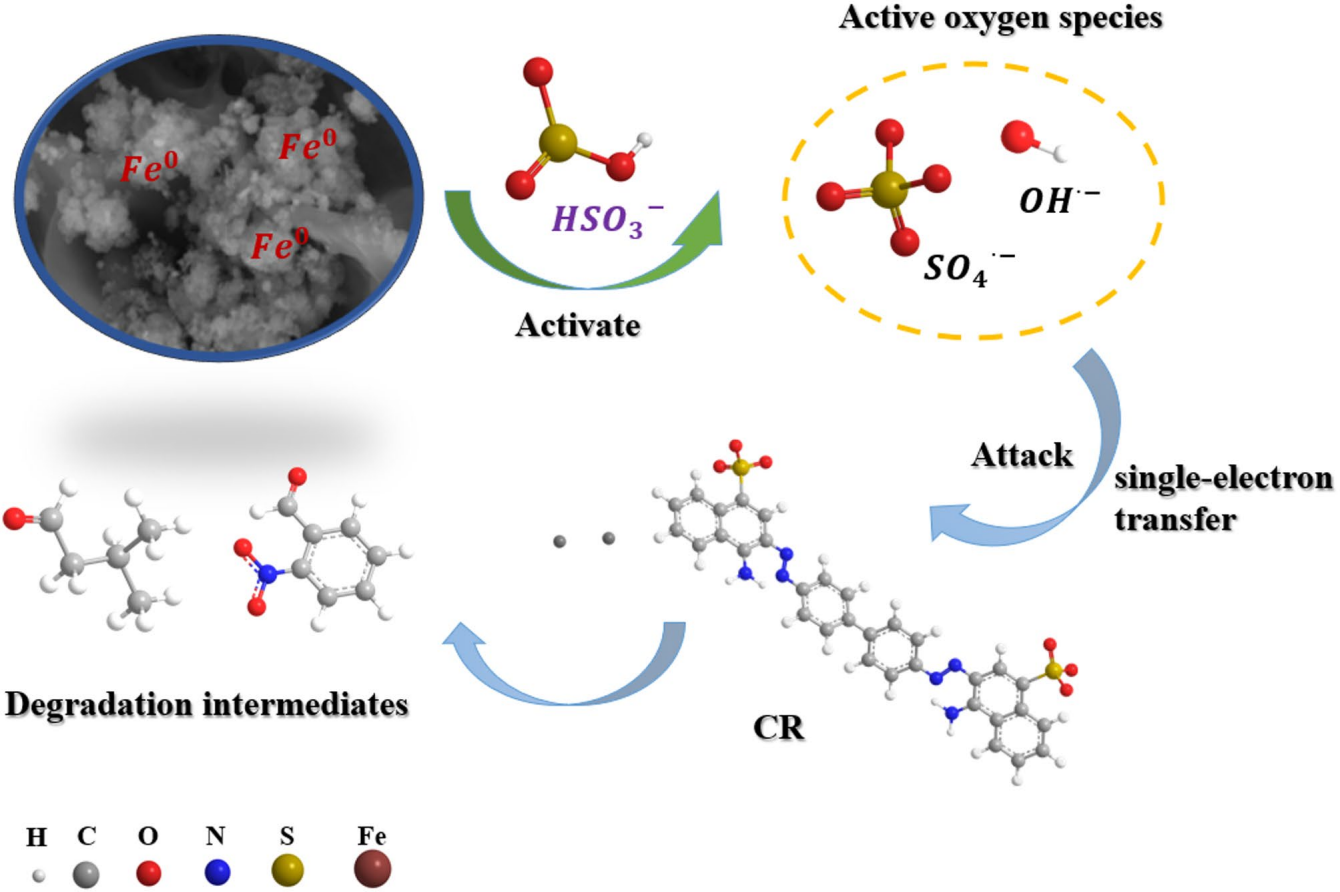
Mechanism of CR degradation by RMAC3-800/BS system.

Based on the experimental results, the proposed mechanism for CR degradation in the RMAC3-800/BS system is shown in Fig. 7. Initially, the CR molecules were adsorbed onto the RMAC3-800 surface. The embedded Fe^0^ then activates BS, generating reactive radicals, mainly SO_4_^•−^ and •OH. These radicals subsequently attacked the adsorbed CR molecules, leading to their oxidative degradation. Quantitative quenching analysis further revealed that SO_4_^•−^ and •OH contributed almost equally to the overall degradation process.

**Fig. 8:**
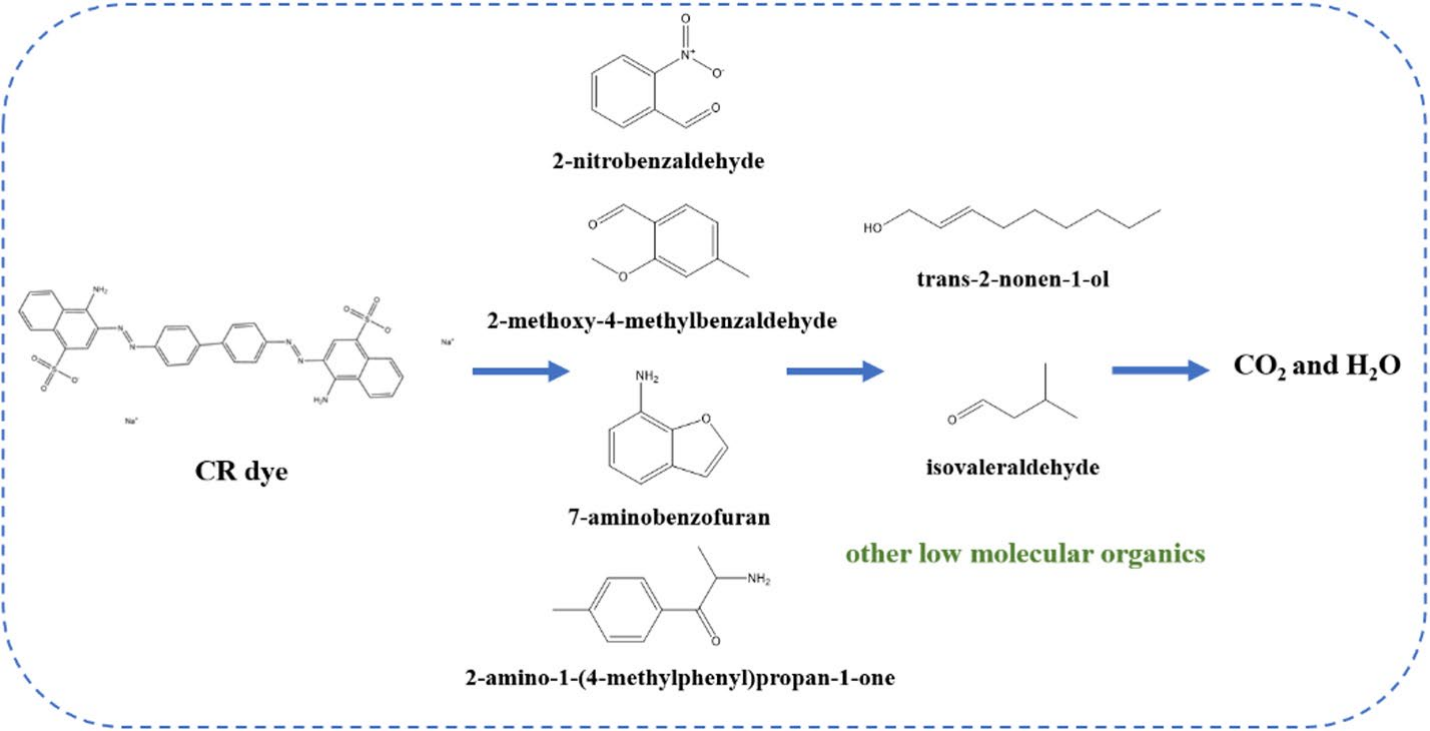
Possible degradation pathways of CR in RMAC3-800/BS system.

To further assess the mineralization performance of the RMAC3-800/BS system, the TOC removal was monitored (Fig. S5). After 60 min, the TOC removal reached 44.2%, indicating partial mineralization of CR and the formation of intermediate species. GC-MS analysis was conducted to identify these intermediates, and six major compounds were detected. The retention times, chemical structures, and molecular weights of these compounds are summarized in Table S3. Based on the identified products, possible degradation pathways of CR were proposed (Fig. 8).

Given that CR contains an azo group (-N = N-) linked to aromatic rings, forming a conjugated chromophore, this structure is particularly prone to oxidative cleavage during degradation. During CR degradation by BS, reactive radicals (SO_4_^•−^ and •OH) disrupt the conjugated system by rapidly attacking the-C-S-, -C-N-, and -N = N- bonds through single-electron transfer reactions^74^. The resulting intermediates are formed via successive electron transfer, bond cleavage, and oxidation^75^.

In the initial stage, SO_4_^•−^ and •OH preferentially attack the -N = N- bond in CR, inducing cleavage, aminonitroxylation, desulfurization, and ring-opening. This leads to the formation of intermediates such as 2-nitrobenzaldehyde, 2-methoxy-4-methylbenzaldehyde, 7-aminobenzofuran, and 2-amino-1-(4-methylphenyl) propan-1-one. These species undergo further transformation through hydrogenation and additional ring-opening reactions, producing smaller aliphatic compounds, including trans-2-nonen-1-ol and isovaleraldehyde. Ultimately, these intermediates are mineralized to CO_2_ and H_2_O. Importantly, no polycyclic aromatic hydrocarbons (PAHs), which are highly toxic byproducts, were detected by GC-MS, indicating a substantial reduction in CR toxicity. Moreover, the identified intermediates exhibit markedly lower toxicity than the parent dye molecule^76^.

### Practical implications and future prospects

Although this study focused on CR as a model pollutant in controlled aqueous systems, the high catalytic activity, stability, and magnetic recyclability of RMAC3-800 highlight its potential for wastewater treatment. Low Fe leaching (< 0.9 mg L^−1^) and stable performance over three consecutive cycles further support its practical feasibility. In addition, a preliminary bench-scale cost analysis estimated an operating cost of ~ 13.94 RMB m^−3^ (≈ 1.95 USD m^−3^) under optimized conditions, with catalyst amortization identified as the dominant contributor, while sodium bisulfite and electricity accounted for minor fractions (Text S3; Tables S4–S5)^77^. These findings demonstrate that RMAC3-800 is an economically viable and recyclable catalytic system. Nonetheless, its efficiency in complex real water matrices (e.g., tap water, industrial effluents, and municipal wastewater) remains to be verified. Therefore, future studies will focus on pilot-scale studies and comprehensive techno-economic evaluations to confirm its applicability under practical conditions.

## Conclusion

In this study, RMAC catalysts were synthesized via an impregnation-co-pyrolysis strategy for bisulfite activation. Among them, RMAC3-800 (CA: RM = 3:1, 800℃) exhibited the best performance, achieving 98.8% CR removal under optimal conditions (5 mM BS, pH = 5, and 80 mg L^−1^ CR). Citric acid acted simultaneously as an acid activator and carbon template, significantly increasing the surface area, porosity, and defect density, thereby enhancing the active site accessibility for CR adsorption and degradation. Mechanistic investigations confirmed that Fe^0^ served as the primary active site, whereas SO_4_^•−^ and •OH were the dominant reactive species. RMAC3-800 maintained > 80% efficiency after three cycles and could be easily recovered magnetically, demonstrating good stability and recyclability. Overall, RMAC3-800 represents a cost-effective and recyclable catalytic system with promising applicability for wastewater treatment and red mud valorization, although further validation in real wastewater is still required.

## Supplementary Information

Below is the link to the electronic supplementary material.

Supplementary Material 1

## Data Availability

The datasets used and/or analysed during the current study available from the corresponding author on reasonable request.
