# Supplementary material for: Citric acid modified red mud for valorization as a sustainable catalyst in bisulfite-activated congo red degradation
Source: Sci Rep. 2025 Oct 21;15:36677. doi: 10.1038/s41598-025-20326-w (PMC12541036; doi:10.1038/s41598-025-20326-w)
Supplement: Supplementary file 1 — Supplementary Material 1 [file 41598_2025_20326_MOESM1_ESM.docx]

**Supplementary material for**

**Citric acid modified red mud for valorization as a sustainable catalyst in bisulfite-activated Congo Red degradation**

**Yonghua Huang ^a^, Cong Zhao ^a^, Shuai Liang ^a^, Zheng Wu ^b^, Daoping Peng ^a,*^, Yao Li ^a,*^, Yun Liu ^c^**

^a^ *School of Environmental Science and Engineering, Southwest Jiaotong University, Chengdu 611756, P.R. China*

^b^ *School of Emergency Management, Xihua University, Chengdu 610039, P.R. China*

*^c^ Sichuan Jiucheng Testing Technology Co, Chengdu 610000, P.R. China*

***** Daoping Peng and Yao Li contributed equally to this manuscript.

*** Corresponding Author：** Daoping Peng, Yao Li

**E-mail:** [pdp0330@swjtu.edu.cn](mailto:pdp0330@swjtu.edu.cn)

**Address:**

Daoping Peng: School of Environmental Science and Engineering, Southwest Jiaotong University, No. 999, Xi’an Road, Pidu District, Chengdu, Sichuan, 611756, P. R. China

**Captions**

**Text S1** Characterization methods.

**Text S2** Analytical methods.

**Text S3** Preliminary Cost Estimation (Bench Scale).

**Fig. S1** XPS spectra of RMACx-y at different mass ratios and pyrolysis temperatures: C 1s (a), O 1s (b); Raman spectra of RMACx-y at different mass ratios and pyrolysis temperatures (c).

**Fig. S2** Effectiveness of RMAC3-800/BS system in degrading CR.

**Fig. S3** Hysteresis loop of RMAC3-800.

**Fig. S4**. Pseudo-first-order kinetic fitting of CR degradation by RMAC3-800 alone, BS alone, and RMAC3-800/BS systems.

**Fig. S5** TOC removal of CR solution in the RMAC3-800/BS system within 60 min reaction time.

**Table S1** BET results of the prepared samples

**Table S2** •OH and SO_4_^•-^ contribution to CR degradation

**Table S3** Degradation intermediates identified by GC-MS

**Table S4**. Batch synthesis cost results.

**Table S5** Operating cost per cubic meter of wastewater.

**Text S1 Characterization methods**

The surface morphology of the samples was examined using a ZEISS Gemini 300 scanning electron microscope (SEM) equipped with an energy-dispersive spectrometer (EDS) for elemental composition and surface distribution analysis. The crystalline structures were characterized by X-ray diffraction (XRD, X'Pert-PRO), and the diffraction patterns were analyzed with MDI Jade 6.0 software. The textural properties, including specific surface area and pore size distribution, were determined from N₂ adsorption-desorption isotherms using the Brunauer-Emmett-Teller (BET) method. X-ray photoelectron spectroscopy (XPS, Thermo Scientific K-Alpha) was employed to investigate surface elemental composition and valence states of RMAC before and after reaction. Raman spectroscopy was used to assess graphitization degree, structural defects, and disorder. Thermogravimetric analysis (TGA) was carried out to evaluate mass changes with temperature or time, providing information on composition and thermal stability. Magnetic properties were measured with a vibrating sample magnetometer (VSM), based on the magnetization response under varying applied magnetic fields.

**Text S2 Analytical methods**

An inductively coupled plasma optical emission spectrometer (ICP-OES, Agilent 7800) was used to monitor the concentration of ferric ions in solution during the reaction. Reactive oxygen species (ROS) were probed through quenching experiments with methanol (MeOH) and tert-butyl alcohol (TBA). MeOH quenches both •OH and SO_4_^•-^, whereas TBA selectively quenches •OH. Under identical experimental conditions, CR removal after 30 min was measured to identify the dominant ROS contributing to degradation. The generation of SO_4_^•-^ and •OH was further verified by electron paramagnetic resonance (EPR) spectroscopy using 5,5-dimethyl-1-pyrroline-N-oxide (DMPO) as the spin-trapping agent. DMPO forms stable adducts with radicals (e.g., DMPO-•OH and DMPO- SO_4_^•-^), whose characteristic signals were detected at 5 and 25 min. Finally, gas chromatography-mass spectrometry (GC-MS) was employed to analyze the reaction solution, identify degradation intermediates, and propose possible CR degradation pathways.

**Text S3 Preliminary Cost Estimation (Bench Scale)**

The cost estimation was based on chemical consumption (citric acid and sodium bisulfite) and electricity usage for calcination and mixing, with red mud considered cost-free as an industrial residue. The analysis followed process-level costing methodologies, adapted to the present bench-scale synthesis. The adopted parameter values were as follows: citric acid price, 5.0 RMB·kg^-1^ (market range 5-7 RMB·kg^-1^, 2024-2025); sodium bisulfite price, 2.2 RMB·kg^-1^ (range 1.9-2.5 RMB·kg^-1^); electricity tariff, 0.770 RMB·kWh^-1^ (Chengdu industrial quotation, March 2025); tube furnace power, 2.0 kW, operated for 2 h per batch; mixing electricity consumption, 0.05 kWh/m^3^; catalyst dosage, 0.5 g L^-1^ with reuse for three cycles; and overall yield, 55.2% (experimentally measured). Costs associated with pH adjustment were neglected due to minimal reagent consumption. Detailed results are presented in Tables S4-S5, and the calculation framework is provided in Eqs. (1)-(5).

$\text{F}\text{urnace electricity consumption }\left( \text{kWh} \right)\text{:}\text{E}_{\text{furnace}}\text{ =}\text{ }\text{P}_{\text{furnace}}\text{×}\text{t}_{\text{h}\text{eat}}\text{ }\text{ }$ (1)

where P_furnace_ is furnace power (kW), t_heat_ is heating duration (h).

$\text{T}\text{otal synthesis cost }\left( \text{RMB} \right)\text{:}\text{C}_{\text{syn,total}}\text{ =}\text{ }\text{C}_{\text{syn,chem}}\text{+}\text{C}_{\text{syn,el}}\text{ }\text{ }$ (2)

where C_syn,chem_ is citric acid cost, C_syn,el_ is electricity cost.

$\text{U}\text{nit catalyst cost (RMB}\text{ g}\text{-1}\text{):}\text{C}_{\text{cat}}\text{ =}\text{ }\text{C}_{\text{syn,}\text{total}}\text{/}\text{m}_{\text{cat,out}}\text{ }\text{ }$ (3)

where m_cat,out_ is catalyst output mass (g).

$\text{T}\text{otal operating cost per m³ (RMB):}\text{C}_{\text{total,1}\text{m}^{\text{3}}}\text{ =}\text{C}_{\text{B}\text{S}\text{,1}\text{m}^{\text{3}}}\text{+}\text{C}_{\text{p}\text{H}\text{,1}\text{m}^{\text{3}}}\text{+}\text{C}_{\text{c}\text{at}\text{,1}\text{m}^{\text{3}}}\text{+}\text{C}_{\text{el,1}\text{m}^{\text{3}}}$(4)

C_BS,1m3_: sodium bisulfite cost (RMB/m³), C_pH,1m3_: pH adjustment cost (RMB/m³, negligible here), C_cat,1m3_: catalyst amortized cost (RMB/m³), C_el,1m3_: reaction electricity cost (RMB/m³).

$\text{y}\text{ =}\text{ }\text{m}_{\text{cat,out}}\text{/(}\text{m}_{\text{RM,in}}\text{+}\text{m}_{\text{C}\text{A,in}}\text{)}$ (5)

where y is overall yield, m_cat,out_ is catalyst output mass (g), m_RM,in_ is red mud input mass (g), m_CA,in_ is citric acid input mass (g).

**Fig S1**


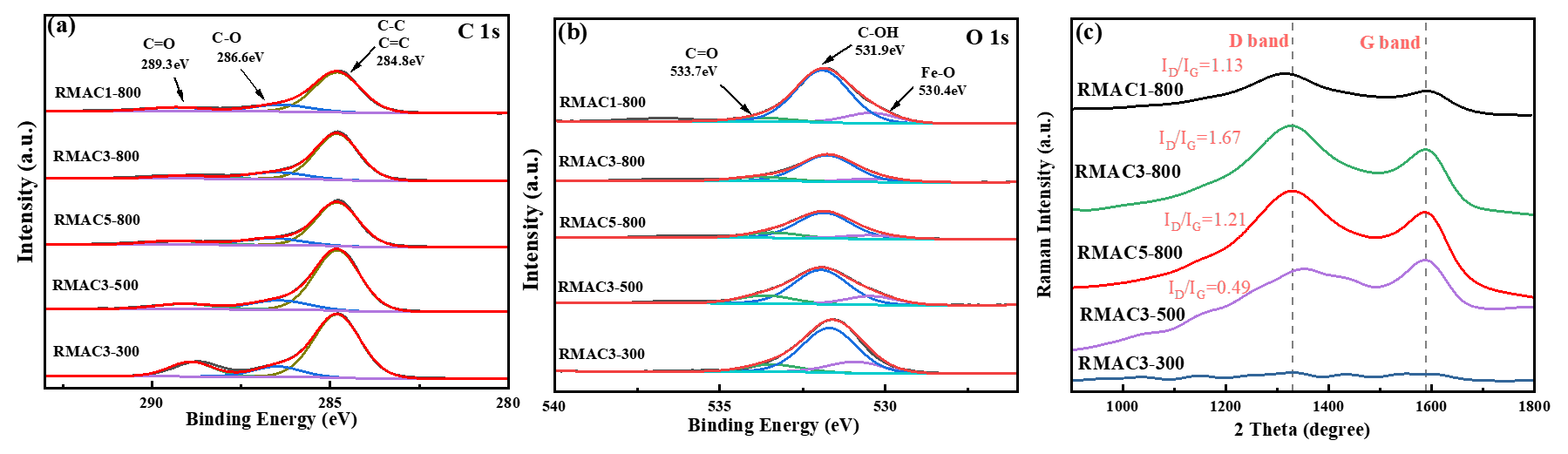


**Fig. S1** XPS spectra of RMACx-y at different mass ratios and pyrolysis temperatures: C 1s (a), O 1s (b); Raman spectra of RMACx-y at different mass ratios and pyrolysis temperatures (c)

**Fig S2**


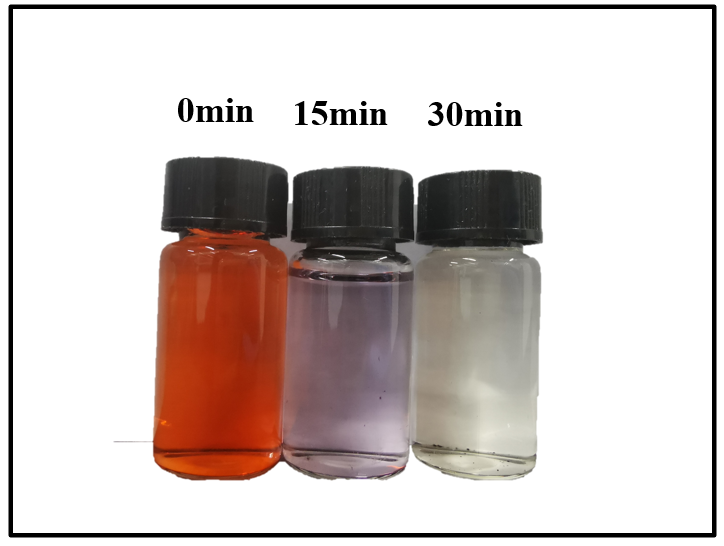


**Fig. S2** Effectiveness of RMAC3-800/BS system in degrading CR

**Fig S3**


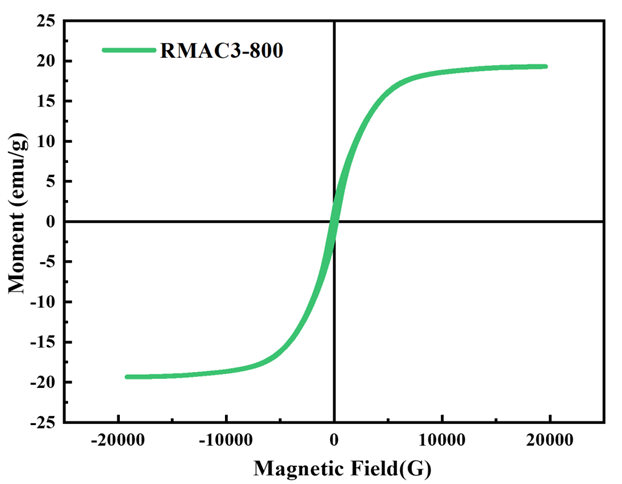


**Fig. S3** Hysteresis loop of RMAC3-800

**Fig S4**


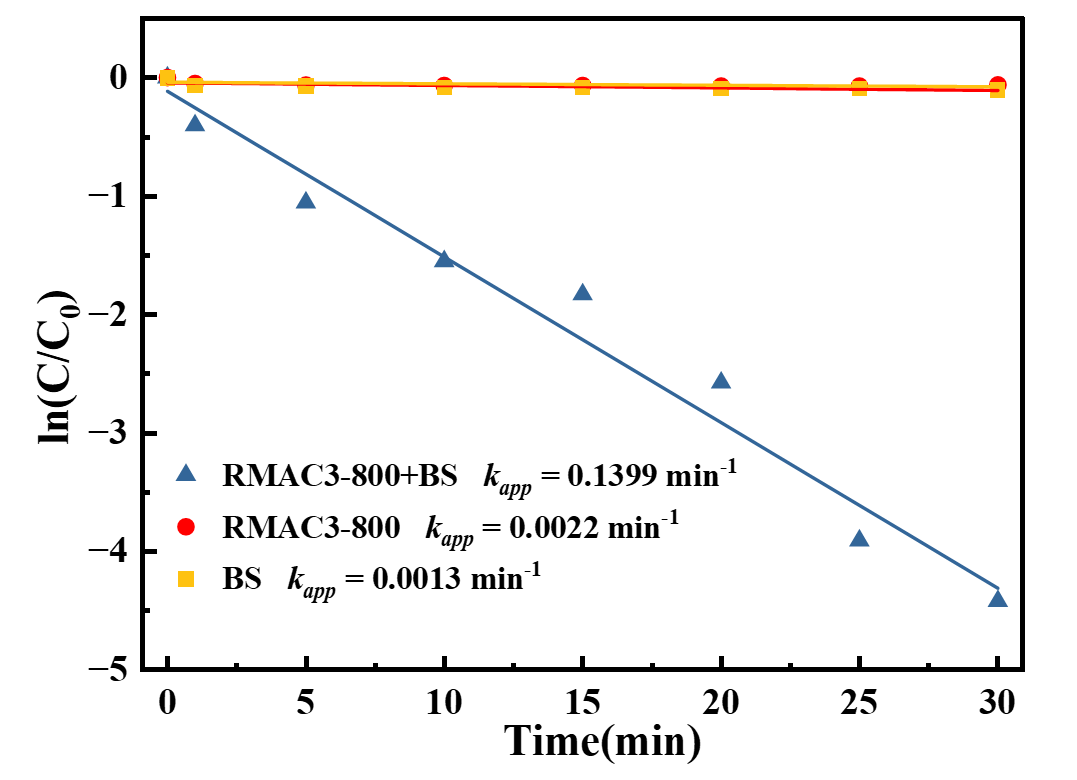


**Fig. S4**. Pseudo-first-order kinetic fitting of CR degradation by RMAC3-800 alone, BS alone, and RMAC3-800/BS systems. Experimental conditions: pH = 5.0, [CR]_0_ = 80 mg L^-1^, RMAC catalyst dosage = 0.5 g L^-1^, [BS] = 5 mM.

**Fig S5**


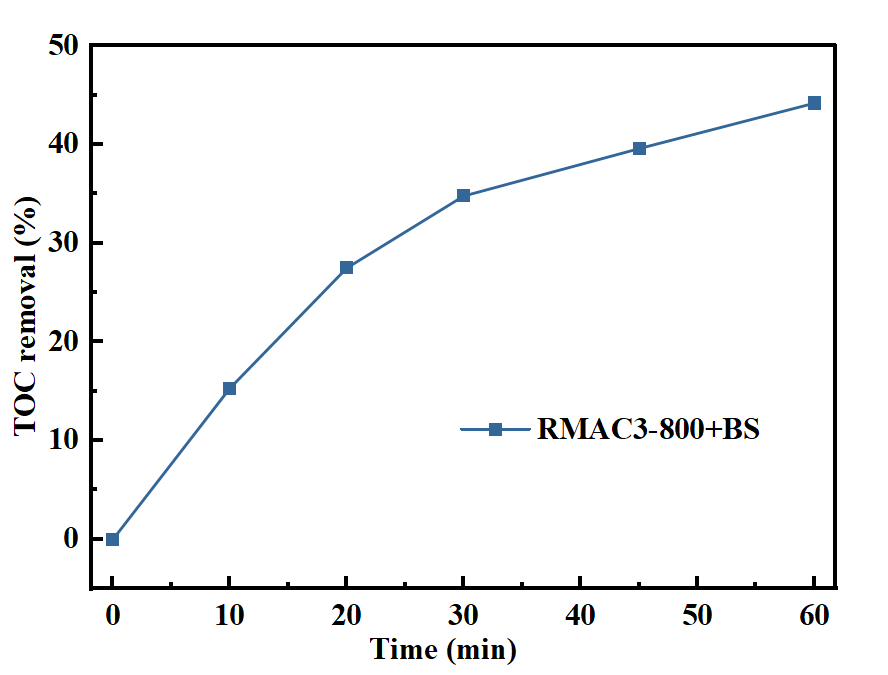


**Fig. S5.** TOC removal of CR solution in the RMAC3-800/BS system within 60 min reaction time. Experimental conditions: pH = 5.0, [CR]_0_ = 80 mg L^-1^, RMAC catalyst dosage = 0.5 g L^-1^, [BS] = 5 mM.

**Table S1**

**Table S1** BET results of the prepared samples

| **Samples** | ***S*_BET_ (m^2^ g^-1^)** | ***V*(cm^3^ g^-1^)** | ***D*_BET_ (nm)** |
| --- | --- | --- | --- |
| RM-800 | 31.16 | 0.076 | 9.76 |
| RMAC3-800 | 116.40 | 0.151065 | 51.912 |

**Table S2**

**Table S2** •OH and SO_4_^•-^ contribution to CR degradation

| Different quenching systems | ∙OH | SO_4_·^-^ | removal/% |
| --- | --- | --- | --- |
| 1. RMAC3-800/BS | √ | √ | 98.8 |
| 2. RMAC3-800/BS +MeOH | |  | 6.67 |
| 3. RMAC3-800/BS +TBA |  | √ | 56.2 |
| Single-system contribution rate | 42.6% | 49.5% |  |
| **Relative contribution rate** | **46.3%** | **53.7%** | **100** |

**Table S3**

**Table S3** Degradation intermediates identified by GC-MS

| Retention time(min) | Chemical structure | Chemical name | Formula | Molecular weight |
| --- | --- | --- | --- | --- |
| 27.848 | 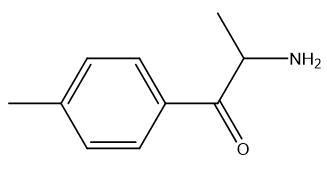 | 2-amino-1-(4-methylphenyl) propan-1-one | C_10_H_13_NO | 163 |
| 5.117 |  | 2-methoxy-4-methylbenzaldehyde | C_9_H_10_O_2_ | 150 |
| 7.131 |  | 7-aminobenzofuran | C_8_H_7_NO | 133 |
| 8.039 |  | 2-nitrobenzaldehyde | C_7_H_5_NO_3_ | 151 |
| 39.450 |  | isovaleraldehyde | C_5_H_10_O | 86 |
| 21.021 |  | trans-2-Nonen-1-ol | C_9_H_18_O | 142 |

**Table S4**

**Table S4** Batch synthesis cost results.

| Overall yield y | Catalyst output (g) | Catalyst cost C_cat_ (RMB g^-1^) |
| --- | --- | --- |
| 0.552 | 44.160 | 0.077 |

**Table S5**

**Table S5.** Operating cost per cubic meter of wastewater.

| BS cost (RMB/m^3^) | Catalyst amortized (RMB/m^3^) | Reaction electricity (RMB/m^3^) | Total (RMB/m^3^) |
| --- | --- | --- | --- |
| 1.145 | 12.757 | 0.039 | 13.941 |

Converted to USD (1 RMB ≈ 0.14 USD): total ≈ 1.95 USD/m^3^; catalyst cost ≈ 0.011 USD g^-1^.
